# A noncanonical role for Jagged1 in endothelial mechanotransduction

**DOI:** 10.1111/febs.70466

**Published:** 2026-02-23

**Authors:** Freddy Suarez Rodriguez, Noora Virtanen, Elmeri Kiviluoto, Rob C. H. Driessen, Feihu Zhao, Carlijn V. C. Bouten, Oscar M. J. A. Stassen, Cecilia M. Sahlgren

**Affiliations:** ^1^ Faculty of Science and Engineering, Cell Biology Åbo Akademi University Turku Finland; ^2^ Turku Bioscience Åbo Akademi University and University of Turku Turku Finland; ^3^ InFLAMES Research Flagship Center Åbo Akademi University and University of Turku Turku Finland; ^4^ Department of Biomedical Engineering Eindhoven University of Technology Eindhoven The Netherlands; ^5^ Institute for Complex Molecular Systems (ICMS), Eindhoven University of Technology Eindhoven The Netherlands; ^6^ Department of Biomedical Engineering, Zienkiewicz Institute for Modelling, Data & AI, Faculty of Science and Engineering Swansea University Swansea UK

**Keywords:** endothelial cells, hemodynamic forces, jagged1, mechanotransduction, notch signaling

## Abstract

The Notch signaling pathway plays a crucial role in regulating endothelial biology. Notch signaling is sensitive to hemodynamic forces and governs mechanically driven cardiovascular development, physiology, and remodeling. However, the mechanisms by which mechanical forces integrate with the Notch pathway remain largely unknown. Here, we uncover a noncanonical role for the Notch ligand protein jagged‐1 (Jagged1) in regulating the activity of mechanosensitive kinases in endothelial cells. We show that shear stress induces expression and relocalization of Jagged1 to cell junctions downstream of flow. *Jagged1* expression under stress demonstrates magnitude dependence, and peaks at 0.8–1 Pa without impacting the Notch‐activation potential of Jagged1. Jagged1 also regulates the activity of mechanosensitive kinases. Deletion of *Jagged1* reduces the activity of vascular endothelial growth factor receptor 2 (VEGFR2) and mitogen‐activated protein kinase (ERK) *in vitro* and diminished ERK activity in zebrafish embryos without affecting canonical Notch signaling. Furthermore, direct physical stimulation of Jagged1 using antibody‐conjugated beads triggers the activation of VEGFR2 and ERK, mediated by Jagged1‐induced proto‐oncogene tyrosine‐protein kinase Src activation. Taken together, we demonstrate a previously unknown noncanonical role for Jagged1 as a regulator of the activity of pathways involved in endothelial mechanotransduction.

## Graphical abstract

The Notch signaling pathway plays a crucial role in regulating endothelial biology. Notch signaling is sensitive to hemodynamic forces and governs mechanically driven cardiovascular development, physiology, and remodeling. This study reveals a non‐canonical role for the Notch ligand Jagged1 in endothelial mechanotransduction. Shear stress modulates Jagged1 expression and subcellular localization. Loss of Jagged1 attenuates mechanotransduction and reduces Src, VEGFR2, and ERK signaling. Direct mechanical stimulation of Jagged1 induces activation of these signaling pathways. These results demonstrate a previously unknown noncanonical role for Jagged1in transducing mechanical cues in the endothelium.

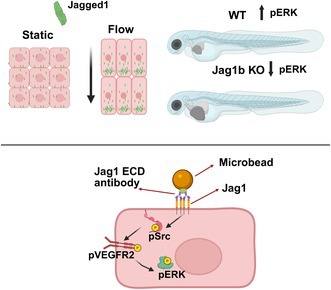

**Abbreviations**
CFD: Computational fluid dynamics
Co‐IP: Co‐immunoprecipitation assay
dpf: Days post fertilization
ECD: Extracellular domain
FSS: Fluid shear stress
HAoECs: Human aortic endothelial cells
hpf: Hours post fertilization
HUVEC: Human umbilical vein endothelial cells
J1ECD ab: Jag1 ECD antibodies
Jag1: Jagged1
KO: Knock‐out
NECD ab: Notch ECD antibodies
NICD: Notch intracellular domain
NTC: nontargeting control
OSI: Oscillatory shear index
PBM: PDZ‐binding motifs
PLA: Proximity ligation assays
rIgG: Recombinant IgG
rNECD: Recombinant Notch ECD
RT: Room temperature
WB: Western blot
WT: Wildtype

## Introduction

Mechanical forces generated by blood flow direct cardiovascular morphogenesis, homeostasis, and disease progression [1, 2]. Endothelial cells lining the lumen of cardiovascular tissues are exposed to fluid shear stress (FSS), which is an essential biophysical signal for vasculogenesis, angiogenesis, arterial specification, vessel maturation, and barrier function [1, 3, 4]. FSS is also a well‐known predictor of atherosclerotic plaque formation, with zones of lower magnitude of FSS and higher oscillatory shear index (OSI) being atheroprone, and those with laminar‐like (low OSI) and higher magnitudes of FSS considered atheroprotective [1, 4, 5]. Physiological FSS varies in different vessels, typically ranging from magnitudes as low as 0.4 Pa to as high as 7 Pa [1, 6, 7]. Endothelial cells express mechanosensors such as the primary cilium, the glycocalyx, Piezo ion channels, the VEGFR2‐VE‐cadherin‐PECAM junctional mechanosensory complex, and PlexinD1, which activate downstream kinases including ERK, AKT, and Src, regulating endothelial cell responses to FSS [8, 9, 10]. Src family kinase occupy a special role in FSS signaling by being activated both upstream and downstream of VEGFR2, aiding in initiating and maintaining signaling activity [8, 11, 12].

The Notch signaling pathway is an essential regulator of cardiovascular development and homeostasis [13, 14, 15]. Notch plays an important role in angiogenesis, arterial specification, endothelial barrier function, cardiac morphogenesis, and arterial remodeling [14, 16, 17, 18, 19, 20]. In canonical Notch signaling, Notch ligands on the membrane of signal‐sending cells bind and activate Notch receptors on signal‐receiving cells. Activation of the receptor requires endocytosis of the ligand after receptor binding [21]. The activated receptor is then cleaved, and its intracellular domain is translocated to the nucleus, where it promotes the transcription of Notch target genes [22]. Notch activity is mechanosensitive, and Notch signaling is essential for the cellular response to hemodynamic stress [13, 23, 24, 25]. Laminar‐like and high magnitude FSS, above 2 Pa, has been shown to enhance Notch1 activity [26, 27], while OSI of high (1.5 Pa) and low (0.4 Pa) magnitudes have been found to stimulate Notch3 and Notch4 activity, respectively [28, 29].

FSS‐induced Notch1 activity in arteries promotes vascular barrier integrity [18] and serves an atheroprotective role in regions exposed to high laminar blood flow [27]. In contrast to Notch1, recent data suggest that the Notch ligand Jagged1 (Jag1) is enriched in pro‐atherogenic zones, of FSS with low magnitude and high OSI [29, 30], and promotes atherosclerosis [29]. However, the exact role of Jag1 in the endothelium remains unclear. Here, we set out to investigated the flow‐responsive nature of Jag1, and its role in endothelial mechanotransduction. We show that magnitude‐specific changes in FSS tune Jag1 expression and localization and identify a noncanonical role of Jag1 in regulating the activity of FSS‐responsive kinases.

## Results

### Shear stress induces changes in Jagged1 expression and localization decoupled from canonical Notch activation

**Fig. 1:**
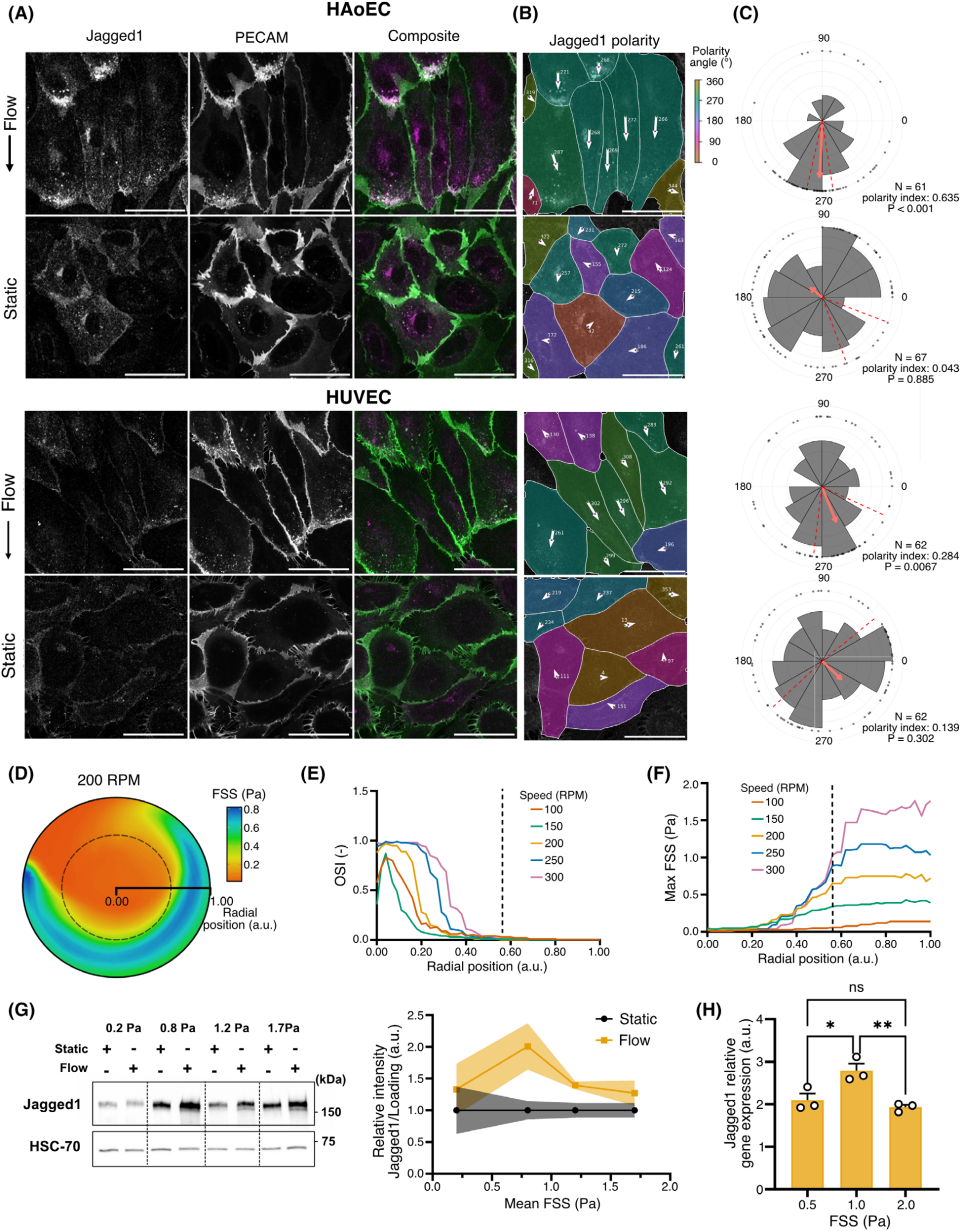
Jagged1 expression and localization in response to FSS. (A) Confocal microscopy images of human aortic endothelial cells (HAoECs) (top) or human umbilical endothelial cells (HUVEC) (bottom) exposed to ~ 1 Pa laminar and continuous Fluid shear stress (FSS) for 24 h in Ibidi microfluidic chips. Jag1 (magenta) demonstrated polarized localization in the direction of flow. PECAM/CD31 (green) was used to denote cell junctions. (B) Analysis of Jag1 polarization in A using PolarityJaM Python API [34]. Mask color represents polarity angle. Vector size and orientation represent magnitude and angle of the polarization. Scale bar for A and B = 50 μm. (C) Merged graph and analysis of independent experiments in PolarityJaM web app. Vector represents the circular mean direction, dotted lines represent the 95% confidence interval. Rayleigh test of uniformity was used to analyze statistical significance. (D) Computational fluid dynamic analysis of the FSS pattern in an orbital shaker system using 6‐well plates. The radial position indicates the radial coordinates of the well, with 0.00 indicating the center of the well and 1.00 the wall of the well. The dotted line separates the wells' outer area with high magnitude laminar flow from the inner area with highly oscillatory flow. Cells were collected from the outer area of the well, from radial position 0.56–1.00. (E) Oscillatory shear index (OSI) was calculated from the simulation and used to determine the zones with the lowest oscillatory flow. (F) Maximum FSS levels were used to determine the magnitude of FSS at each speed (RPM). The dashed line in E and F indicates radial position 0.56 equivalent to the dotted line in D. (G) Western blot (WB) analysis of Jag1 protein levels in HUVECs exposed to different magnitudes of FSS using our orbital shaker system. The values are presented relative to the corresponding static control. (H) Q‐PCR analysis of Jag1 gene expression levels in HUVECs exposed to laminar and continuous FSS of different magnitudes for 24 h in Ibidi microfluidic chips. All experiments were performed three times; for H, this included three technical replicates within each experiment. The levels are presented as the mean of each replicate relative to their corresponding control + standard error of the mean (SEM). For WBs HSC‐70 was used as a loading control. P‐values were obtained with PolarityJAM (C) after Rayleigh test or with GraphPad Prism after one‐way ANOVA (H). Significance is indicated as: ns *p* > 0.05, * *p* < 0.05, ** *p* < 0.01, Arbitrary units are indicated as (a.u.).

We have previously demonstrated that FSS induces clustering of Jag1 into submembranous vesicles, enhances Jag1 endocytosis and Jag1‐induced Notch activation in VSMCs and reporter cells adjacent to the endothelium [31, 32]. Previous data have shown that Notch1, the main receptor expressed in endothelial cells [33], polarizes in the direction of flow [27, 34]. Here, we analyzed the relocalization of Jag1 in response to flow. Surprisingly, in addition to the contralateral localization needed for Jag1‐Notch transactivation, FSS also induced a significant Jag1 polarization in the direction of flow in both human aortic endothelial cells (HAoECs) and human umbilical vein endothelial cells (HUVEC) (Fig. 1A–C) exposed to ~ 1 Pa for 24 h.

**Fig. 2:**
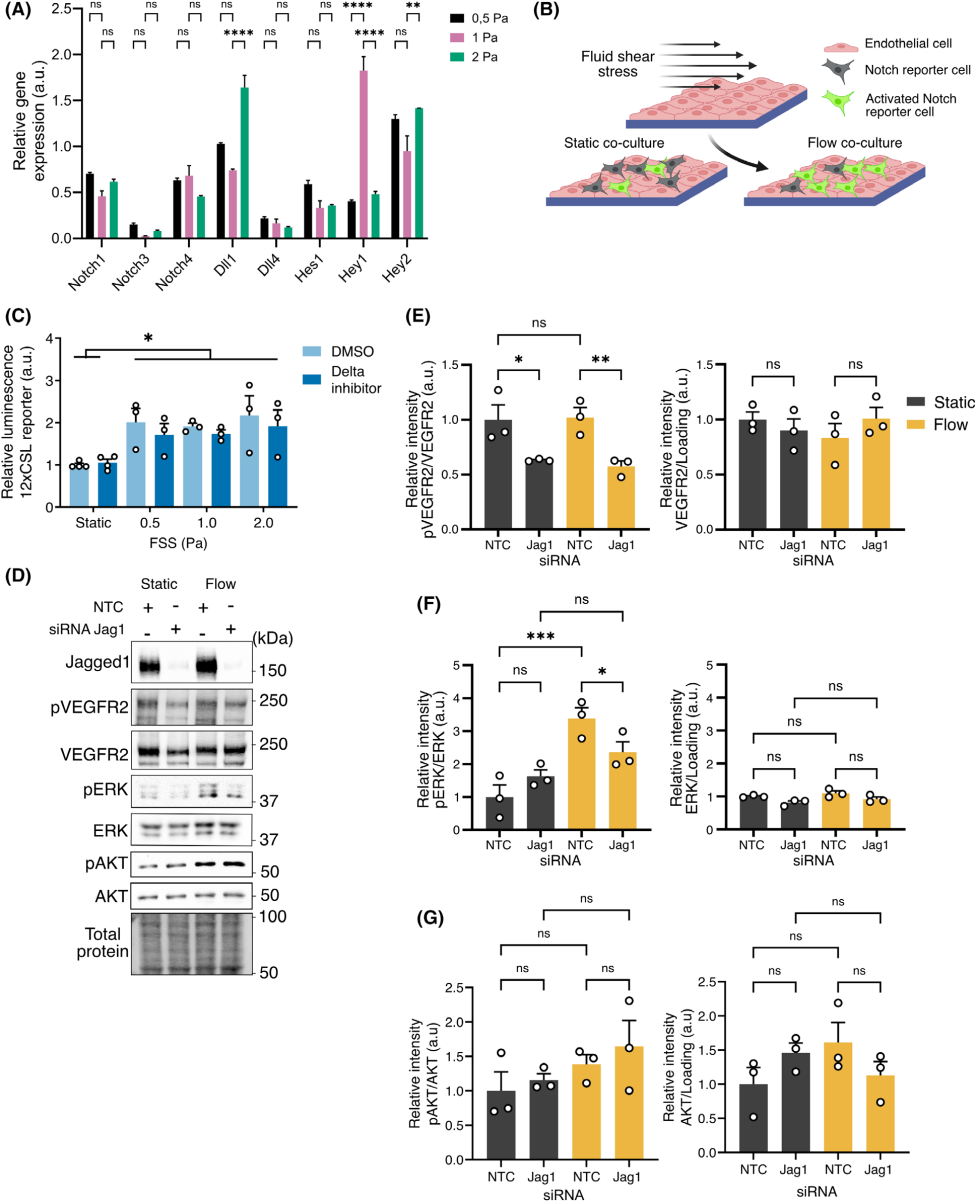
Jag1 is decoupled from its transactivation potential and is necessary for shear stress‐linked kinase activity. (A) Q‐PCR analyses of expression of Notch ligands and receptors in human umbilical vein endothelial cells (HUVECs) exposed to 0.5, 1.0, and 2.0 Pa of laminar and continuous fluid shear stress (FSS) for 24 h in Ibidi Chips. The experiment was performed three times with three technical replicates within each experiment. (B) Schematic of the experiment using Notch reporter cells cultured on HUVECs exposed to shear. (C) Notch activity in 12xCSL‐Luciferase expressing HEK293‐FLN1 cells co‐cultured with HUVECs exposed to different magnitudes of FSS. Delta signaling was inhibited by the fucose analog 6‐alkenyl‐fucose. (D) Western blot (WB) of the phosphorylation and protein expression levels of VEGFR2, ERK, and AKT in HUVECs exposed to 0.8 Pa of Fluid shear stress (FSS) in the orbital shaker. Cells were silenced for 48 h with siRNA nontargeting control (NTC) or siRNA Jag1 before exposure to FSS for 24 h. Quantification of three independent experiments (*n* = 3) is found in (E) for VEGFR2 and phospho‐VEGFR2 (Y1175), (F) for ERK and phospho‐ERK (Y204/Y187), and (G) for AKT and phospho‐AKT (S473). *P*‐values were obtained with GraphPad Prism after two‐way ANOVA with Tuckey's tests (A, C) or Fisher's LSD test (E, F, G). The levels are presented as the mean of each replicate relative to their corresponding control + standard error of the mean (SEM). Significance is indicated as: ns *p* > 0.05, * *p* < 0.05, ** *p* < 0.01, *** *p* < 0.001, **** *p* < 0.0001. Arbitrary units are indicated as (a.u.).

Notably, while Jag1 expression and Notch1 activity increase in cells exposed to FSS [26, 27, 29, 32, 35], their response to changes in FSS magnitude differs. Notch activation has been found to increase with the magnitude of the FSS beyond 2 Pa [26, 27]. In contrast, Jag1 expression was reported to be the highest at low magnitudes of FSS (0.4 Pa) [29]. Given the importance of FSS magnitude, we evaluated the effect shear stress magnitude on the expression of Jag1 and other components of the Notch pathway. To this end, we used an orbital shaker to complement the microfluidic chips and obtain sufficient material for further biochemical analysis. We utilized computational fluidic dynamics to characterize FSS magnitude and OSI in a six‐well cell culture plate (Fig. 1D) to be able to isolate the cells exposed to laminar‐like flow with low OSI (Fig. 1E) and a specific magnitude of FSS (Fig. 1F). Using this system, we confirmed that FSS induces the highest Jag1 expression at low magnitudes of FSS, with protein levels decreasing at 1.2 Pa and beyond (Fig. 1G) in line with what has been reported [29]. Additionally, we reveal that protein levels initially peaked at approximately 0.8 Pa before decreasing. To assess whether FSS affected Jag1 gene expression, we analyzed mRNA levels of the main ligands and receptors (Jag1, Dll4, Notch1 and Notch4) expressed in endothelial cells through qPCR of cells flowed using Ibidi® microfluidic chips. Jag1 expression peaked at 1 Pa (Fig. 1H), further corroborating our results. The expression of Notch receptors did not change significantly at the magnitudes evaluated (Fig. 2A).

The reduction in Jag1 expression below 2 Pa (Fig. 1G,H) [29] is in discrepancy with the continuous increase in Notch activity reported by the literature for FSS above 2 Pa [26, 27]. We next assessed how different FSS magnitudes influenced Jag1 signaling potential by co‐coculturing endothelial cells exposed to FSS together with Notch reporter cells (Fig. 2B) [31]. As endothelial cells also express Dll4, we inhibited Delta signaling using fucose analogs [36] to specifically evaluate the effect of FSS on Jag1‐mediated Notch activation in reporter cells. In agreement with our previous data [31], we found that FSS enhanced Jag1‐mediated Notch activation. However, the levels of endothelial cell‐induced activation of Notch in the reporter cells remained constant regardless of the magnitude of FSS stress tested (Fig. 2C). This contrasts with the FSS magnitude‐dependent Jag1 expression levels (Fig. 1G,H) [29], indicating that FSS‐regulated Jag1 levels may serve unique functions in endothelial cells unrelated to Notch activation. Even though endothelial‐specific knock‐out of Jag1 leads to embryonic lethality at E10.5 [37], Jag1 is considered a weak and redundant Notch activator in endothelial cells [14, 38, 39]. Our results further suggest an uncoupling of Jag1 expression and Notch activation, consistent with their distinct roles in various vascular processes.

### Jag1 deletion impairs VEGFR2 and ERK activity

We next evaluated the involvement of Jag1 in endothelial mechanotransduction. FSS is sensed by mechanoreceptors which activate downstream kinase signaling to regulate endothelial cell function in the hemodynamic environment [1, 4, 5]. We silenced Jag1 using siRNA and assessed the activity of FSS‐responsive kinase activity in endothelial cells exposed to 0.8 Pa shear stress for 24 h in full media including growth factors (Fig. 2D). The phosphorylation levels of VEGFR2 were reduced in both static and flow conditions upon silencing Jag1 (Fig. 2D,E). Jag1 silencing also decreased the phosphorylation of ERK kinase induced by FSS (Fig. 2D,F) but caused no significant changes in the phosphorylation or total expression levels of AKT (Fig. 2D,G).

**Fig. 3:**
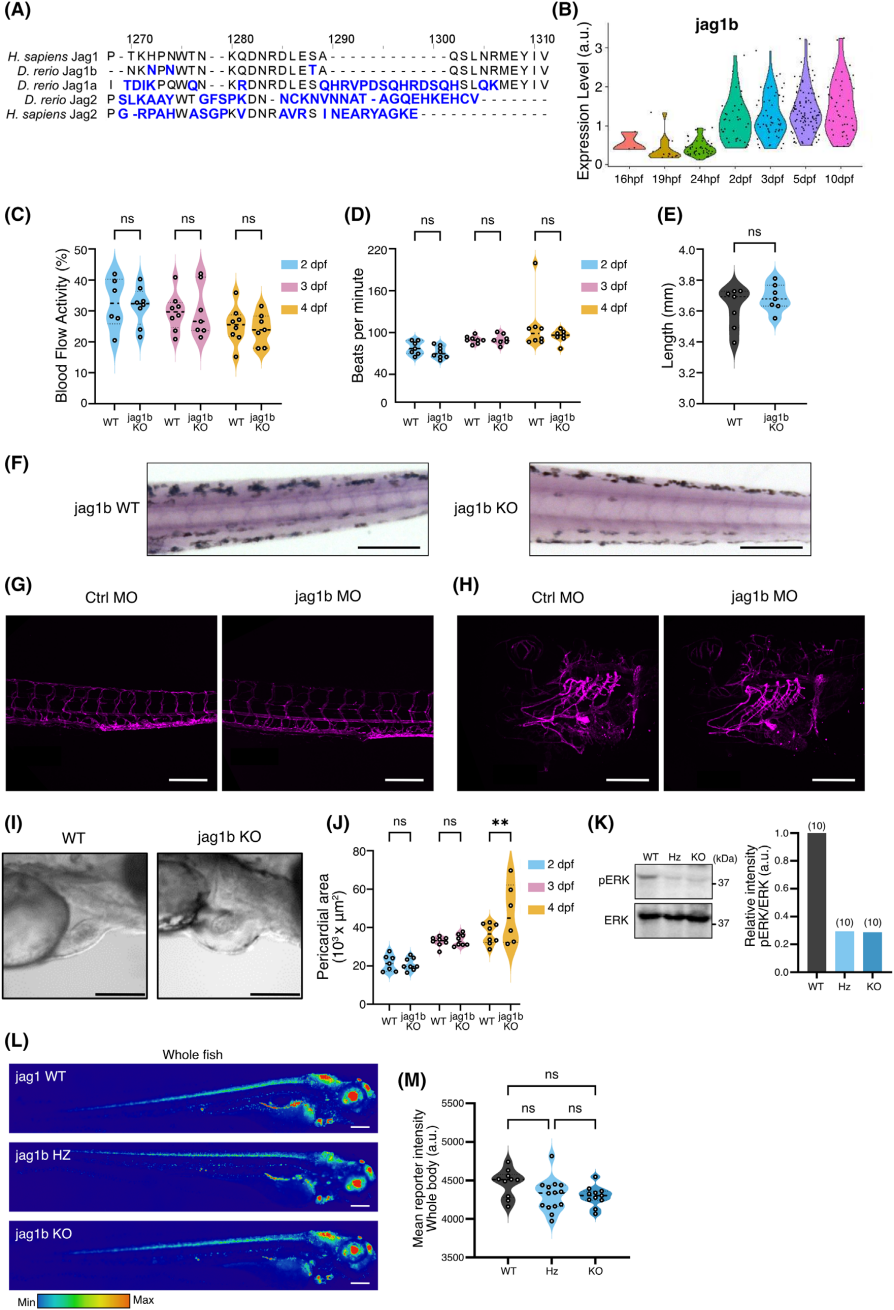
Jag1b deletion induces pericardial edema and reduced ERK activity in zebrafish embryos. (A) Zebrafish (D. rerio) express two orthologs of Jag1, Jag1a and Jag1b. Jag1b shares the human sequence with the last 10 residues at the last N terminus, including the PDZ‐binding motif. Sequences were obtained from UniProt, and the multiple alignment analysis was performed in clustal omega. (B) Gene expression analysis of data from the Zebrahub database of cells expressing the endothelial cell (EC) marker fli1a, the blood vascular EC kdrl and jag1b (fli1a > 1, kdrl > 1 and jag1b > 0). (C) blood flow activity and (D) heart rate as beats per minute of jag1 wildtype (WT) (*n* = 6, 8 and 8 respectively) and jag1b knock‐out (KO) zebrafish embryos at 2, 3, and 4 post fertilization (dpf) (*n* = 8, 7, and 7 respectively). (E) Length in mm of jag1WT and jag1bKO zebrafish embryos at 4 dpf (*n* = 7). (F) Bright‐field images of alkaline phosphatase staining of the vasculature of jag1WT and jag1bKO zebrafish embryos at 4 dpf (*n* = 2). (G, H) Max intensity projection of confocal images of cranial (G) and abdominal (H) vasculature in zebrafish at 4 dpf (*n* = 2) (magenta represents EC marker kdrl). (I) Representative microscopy images of the developing heart in wildtype (WT) and Jag1b knock‐out (KO) zebrafish at 4 dpf. jag1bKO zebrafish show signs of pericardial edema. (J) Quantification of the pericardial area of WT at 2, 3 and 4 dpf (*n* = 7, 8 and 8 respectively), and homozygous Jag1 knock‐out (KO) at 2, 3 and 4 dpf (*n* = 8, 8 and 6 respectively). (K) ERK activity, measured as the ratio of phospho‐ERK (Y204/Y187) over total ERK, was measured in protein lysates of WT (*n* = 10), heterozygous (Hz) (*n* = 10) and KO (*n* = 10) embryos by WB. (L) Fluorescence images of Notch activity (TP1:H2B‐mCherry) in TP1:H2B‐mCherry (Jag1 (WT); TP1:H2B‐mCherry/jag1bHz and TP1:H2B‐mCherry/jag1bKO) zebrafish at 4 dpf. (M) Quantification of Notch activity in the whole fish of WT (*n* = 10), Hz (*n* = 14) and KO (*n* = 11) was performed by measuring fluorescent intensity. All scalecale bars represent 200 μm. The levels are presented as the mean of each replicate relative to their corresponding control + standard error of the mean (SEM). *P*‐values were obtained with GraphPad Prism after two‐way anova with Fisher's LSD test (C–D), unpaired *t*‐test (E) or one‐way ANOVA with Tukey's test (M). Significance is indicated as: ns *p* > 0.05, ** *p* < 0.01.

To test whether Jag1 also affected kinase signaling *in vivo*, we utilized stable Jag1b KO zebrafish (*Danio rerio*) embryos. The zebrafish cardiovascular system begins to develop during the first 24 h postfertilization (hpf) and the onset of flow occurs at 25–26 hpf [40]. Notch activity has been shown to be induced by flow in the vasculature at 2 days postfertilization (dpf) [41]. Zebrafish express two forms of Jag1: jag1a and jag1b. Whereas the extracellular and intracellular domains of jag1b show high similarity to human Jag1, the intracellular domain of jag1a only has 30% similarity with substantial variations in the PDZ‐binding motifs (PBM) that mediate protein–protein interactions (Fig. 3A). jag1b is expressed as early as 16 hpf with an increase in expression after 2 dpf (Fig. 3B). jag1bKO embryos did not show any differences in blood flow, heart rate, growth at 2, 3, and 4 dpf (Fig. 3C–E) nor in vascular architecture as determined by alkaline phosphatase staining at 4 dpf (Fig. 3F). To further assess the impact of jag1b deletion on vascular morphology we knocked down jag1b in endothelial reporter zebrafish, Tg(kdr:EGFP)s843, using morpholinos. We did not observe any gross differences in vascular morphology between control and jag1b knockdowns (Fig. 3G,H). However, the jag1bKO zebrafish demonstrated pericardial edema at 4 dpf (Fig. 3I,J), in line with data on compound jag gene knockdowns demonstrating cardiac outflow tract defects and pericardial edema [42]. Pericardial edema is associated with VEGF inhibitors‐induced cardiovascular toxicity [43] and inhibition of the ERK activator MEK1 has also been shown to induce outflow tract blockage in the heart with concomitant pericardial edema [44, 45, 46].

We next analyzed kinase activity in lysates from WT, jag1b heterozygous, and jag1bKO zebrafish embryos at 4 dpf. The VEGFR2 antibody was incompatible with zebrafish, but ERK activity was significantly reduced in the heterozygote and jag1bKO compared to WT embryos (Fig. 3K). Since both canonical and noncanonical Jag1 signaling may affect ERK activity [47, 48], we crossed the jag1bKO fish with the tg(*TP1:H2B‐mCherry*) Notch reporter line [49] to analyze Notch activity. We did not observe any significant changes in Notch signaling activity upon deletion of jag1b (Fig. 3L), measured by the fluorescence reporter intensity in the whole fish (Fig. 3M). Taken together, the data suggest that Jag1 may play a noncanonical role in regulating the activity of mechanosensitive kinases in endothelial cells.

### Physical stimulation of Jagged1 induces VEGFR2 activity

**Fig. 4:**
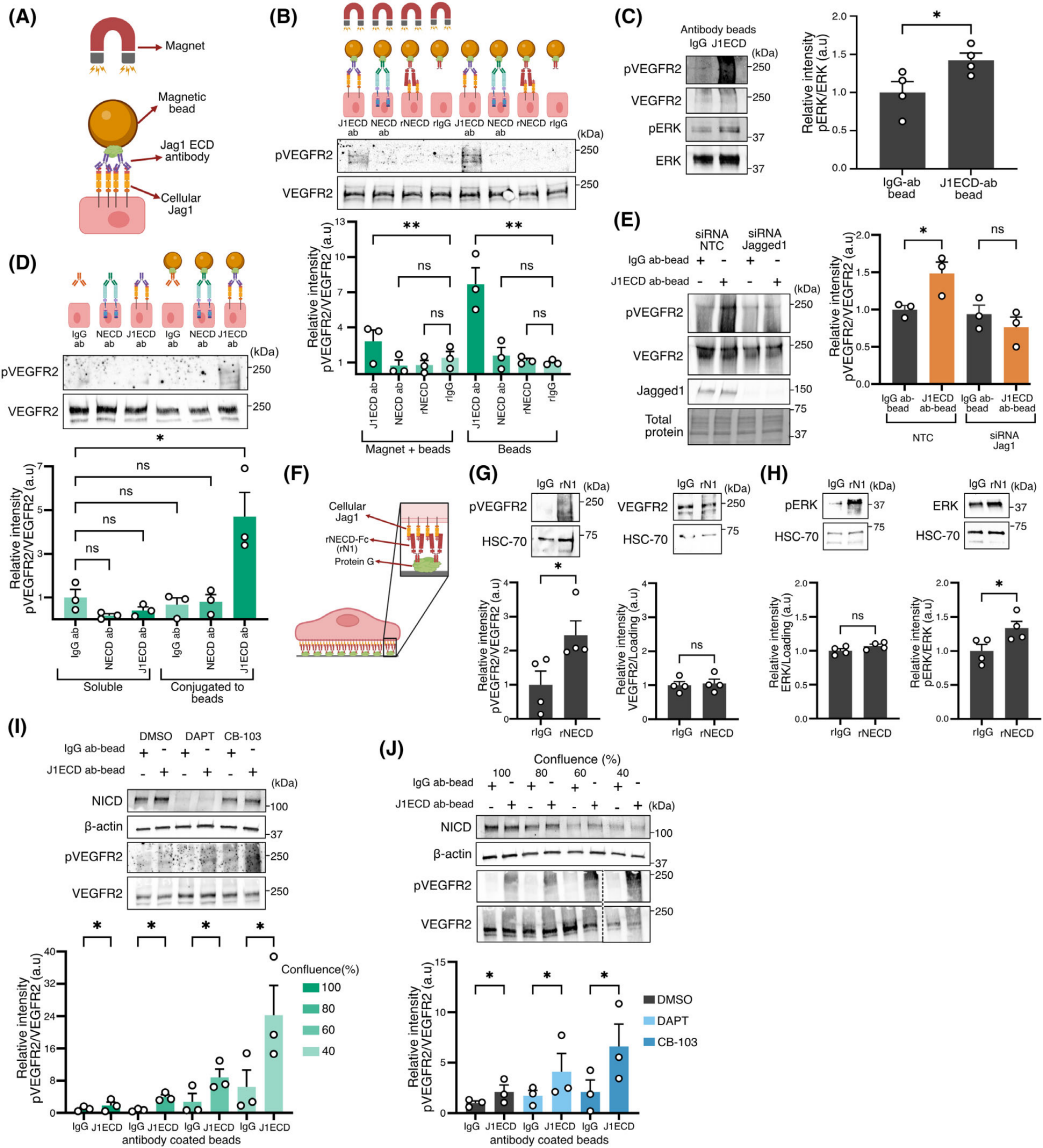
Mechanical stimulation of Jag1 activates shear stress‐related kinases independently of Notch. (A) Schematic illustration (not to scale) of the magnetic bead experiment: human umbilical vein endothelial cells (HUVECs) were incubated with protein A/G magnetic beads coated with Jag1 extracellular domain (ECD) antibodies (J1ECD ab) or other antibodies not shown in the schematic (Notch ECD antibodies (NECD ab), recombinant Notch ECD (rNECD), or recombinant IgG (rIgG)) for 15 min and then exposed to a magnet or incubated with the cells for a total of 30 min. (B) Western blot (WB) and analysis (below) of VEGFR2 and phosphorylated VEGFR2 (Y1175) in HUVECs treated with magnetic beads coated with J1ECD ab, NECD ab, rNECD, or rIgG. VEGFR2 phosphorylation was induced only in cells incubated with magnetic beads coated with the Jag1ECD antibody. (C) WB analysis of phosphorylated ERK (Y204/Y187) and total ERK levels in HUVECs incubated with protein A/G magnetic beads coated with J1ECD‐ab or IgG antibody control (IgG‐ab bead). (D) WB analysis of VEGFR2 and phosphorylated VEGFR2 (Y1175) in HUVECs treated with soluble IgG ab, NECD ab or J1ECD ab, or conjugated to magnetic beads. J1ECD ab‐coated beads but not soluble Jag1 antibodies induced VEGFR2 phosphorylation. (E) WB analysis of VEGFR2 and phosphorylated VEGFR2 (Y1175) in control and Jag1‐silenced HUVECs stimulated by J1ECD ab‐ or IgG ab‐coated beads. siRNA‐mediated silencing of Jag1 inhibited phosphorylation of VEGFR2 induced by the J1ECD ab‐coated beads. (F) Schematic illustration of experimental setup with immobilized recombinant Notch extracellular peptides (rNECD): HUVECs were cultured for 6 h on top of rIgG or rNECD immobilized to protein G‐coated culture dishes. (G) WB analysis of phosphorylated VEGFR2 (Y1175) and total VEGFR2 and (H) phosphorylated ERK (Y204/Y187) and total ERK levels in HUVECs cultured on rIgG or rNECD. (I) WB analysis of phosphorylated VEGFR2 (Y1175) and total VEGFR2 in HUVECs treated with J1ECD ab‐ or IgG ab‐beads in the absence and presence of Notch pathway inhibitors. Pretreatment with Notch inhibitors DAPT and CB‐103 did not reduce the Jag1‐mediated VEGFR2 response. Values were normalized to the IgG ab‐bead control. (J) WB analysis of phosphorylated VEGFR2 (Y1175) and total VEGFR2 in HUVECs of different confluences treated with J1ECD ab‐ or IgG ab‐coated beads. J1ECD ab‐coated beads induced VEGFR2 activation did not decline with decreasing confluence in HUVECs. The values were normalized to the IgG ab‐coated bead control for each confluence. The levels are presented as the mean of each replicate relative to their corresponding control+ SEM. HSC‐70 and whole cell lysates were used as a loading control. All experiments were performed three times. *P*‐values were obtained with GraphPad Prism after two‐way ANOVA with Bonferroni correction (B), Dunnett's test (C) or Fisher's LSD test (D–F). Significance is indicated as: ns *p* > 0.05, * *p* < 0.05, ** *p* < 0.01. Arbitrary units are indicated as (a.u.).

Since Jag1 depletion impaired kinase activity, we next examined whether Jag1‐mediated kinase activity could be induced by direct stimulation of Jag1. To this end, we first applied tensional force on Jag1 using 1 μm paramagnetic beads coated with an antibody that recognizes the extracellular domain of Jag1 (J1ECD ab) (Fig. 4A). A similar system has been used to evaluate the mechanosensitivity of proteins involved in FSS induced signaling [50, 51]. Antibody validation of the J1ECD ab using Jag1 wild‐type and knockout cells is presented in Fig. S1. We exposed endothelial cells to the beads for 15 min, followed by an additional 15 min of exposure to a magnetic force field. VEGFR2 activation was increased in endothelial cells exposed to J1ECD ab‐coated beads but not in those exposed to NECD ab‐, rNECD‐, or rIgG‐coated beads (Fig. 4B,C). Exposure to J1ECD ab‐coated beads also induced ERK activation (Fig. 4C).

The presence of the magnetic field resulted in only a minor increase in Jag1‐induced VEGFR2 activation (Fig. 4B), indicating that the mechanical force might not be necessary to induce VEGFR2 activity in response to Jag1 stimulation. However, exposing cells to soluble antibodies did not induce VEGFR2 activation, indicating that a physical component is necessary for Jag1‐induced VEGFR2 activation (Fig. 4D). This finding is consistent with observations made for VE‐Cadherin [52] and integrins [53, 54, 55], where clustering induced by antibody‐coupled microbeads, without further force, has been shown to induce signal activation. To further confirm the involvement of Jag1 in VEGFR2 activation, we silenced Jag1 in the endothelial cells. siRNA‐mediated Jag1 knockdown prevented VEGFR2 phosphorylation by the J1ECD ab‐coated beads (Fig. 4E).

While rNECD‐coated beads were expected to interact and stimulate Jag1, exposing cells to these beads for 30 min was insufficient to produce a detectable increase in VEGFR2 phosphorylation (Fig. 4D). We also tested whether cells plated on top of immobilized rNECD could activate phosphorylation of VEGFR2 and ERK (Fig. 4F). Increase in phosphorylation was indeed detected but only after 6 h of exposure (Fig. 4G,H). These findings align with previous reports, which show that beads coated with the recombinant Jag1 ligand induce Notch activation after days of incubation [56, 57]. This longer time requirement may be due to the low affinity of the interaction between Jag1 ligands and Notch receptors compared to that of monoclonal antibodies.

To assess whether Jag1‐induced VEGFR2 activation required canonical Notch activity, we treated endothelial cells with Notch inhibitors. The γ‐secretase inhibitor DAPT was used to prevent Notch cleavage and consequently inhibit canonical [58] and noncanonical cortical Notch activation [18]. The more recent inhibitor, CB‐103, was used to inhibit Notch transcriptional activation [59]. We cultured the cells in the presence of the inhibitors for 24 h before exposing them to beads, to ensure efficient Notch inhibition. Neither inhibition of Notch cleavage nor transcriptional activity prevented VEGFR2 activation induced by the J1ECD ab‐coated beads (Fig. 4I). Since canonical Notch activity requires cell–cell contact, seeding the cells at lower confluences reduces Notch activity. We further demonstrated that lowering the confluence of the cultures did not decrease VEGFR2 activation (Fig. 4J). To verify that DAPT treatment and reduced confluence lead to reduced Notch intracellular domain (NICD) levels, we blotted the samples for NICD (Fig. 4I,J). As expected, NICD levels were reduced in the DAPT‐treated and low confluency samples, but not in the CB‐103‐treated samples where transcriptional activity but not cleavage was inhibited. Taken together, the data indicate that Jag1‐mediated VEGFR2 activation does not require canonical Notch activity and is independent of NICD levels.

### Jag1‐mediated signaling requires Src activity

**Fig. 5:**
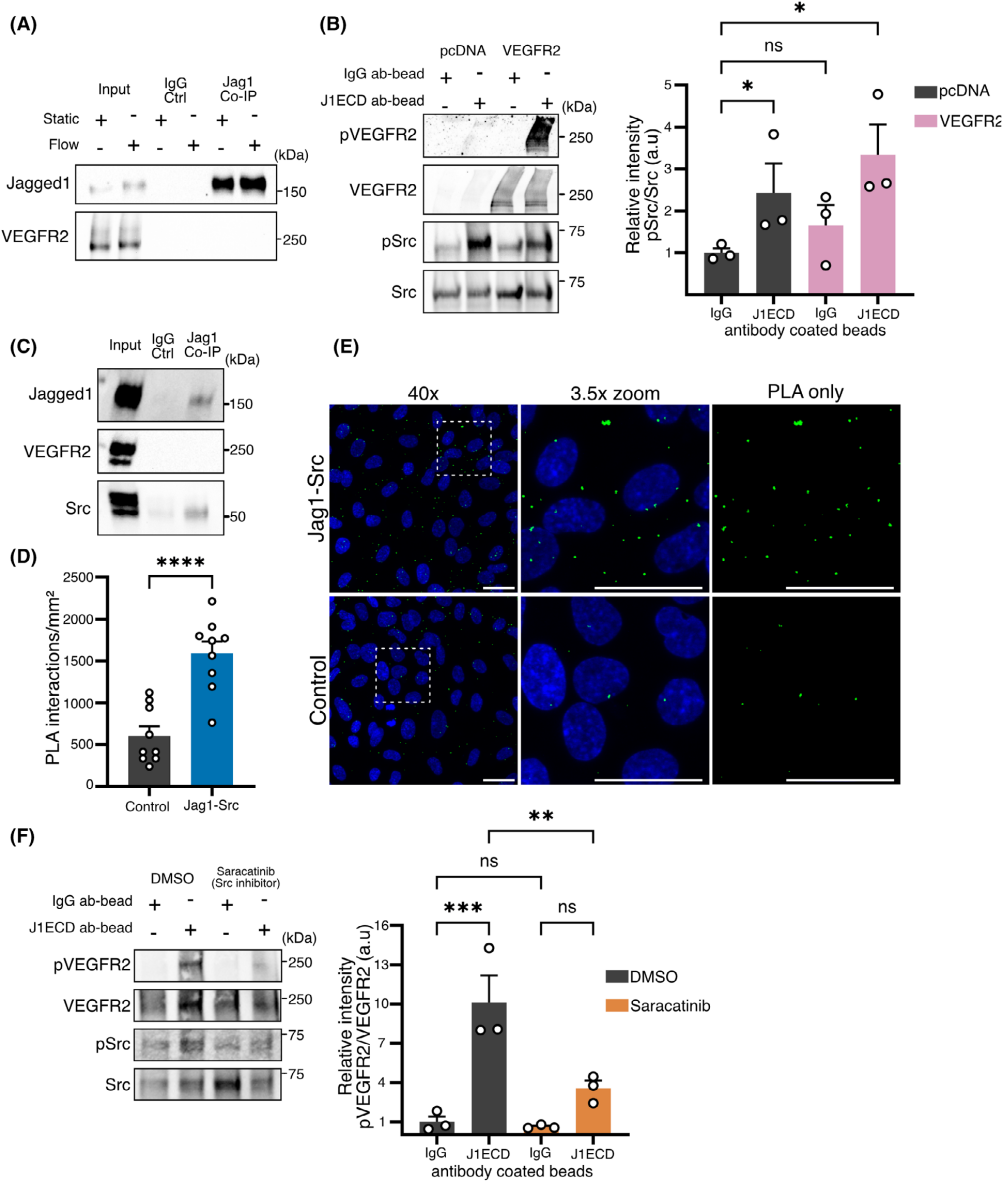
Jag1‐mediated VEGFR2 activation requires Src. (A) Jag1 Co‐Immunoprecipitation (Co‐IP) analysis by western blot (WB), of VEGFR2 in human umbilical vein endothelial cells (HUVECs) cultured in static conditions or exposed to flow (0.8 Pa Fluid shear stress (FSS)) using the orbital shaker system. No VEGFR2 was detected in Jag1 Co‐IP samples. (B) WB analysis of total VEGFR2 and phosphorylated VEGFR2 (Y1175), and total Src and phosphorylated Src (Y416) in COS‐7 stimulated with Jag1 extracellular domain (J1ECD) antibodies (ab)‐ or IgG ab‐coated beads after being transfected with plasmid control (pcDNA) or the VEGFR2 expression plasmid. Stimulation of COS‐7 cells with J1ECD ab‐coated beads induced Src and VEGFR2 activation. Src was activated in the absence of VEGFR2. The values were normalized to the IgG ab‐bead control. (C) Jag1 Co‐IP analysis by WB of VEGFR2 and Src in HUVECs cultured in static conditions. Src but not VEGFR2 are detected in Jag1 Co‐IP samples. (D) Automated quantification of the average proximity ligation assay (PLA) dot count per area (mm^2^) on 18 images from three individual experiments using cellprofiler. PLA was performed in HUVECs cultured in static conditions. (E) Representative confocal microscopy images of the PLA experiment, (DAPI in blue and the PLA signal in green). Scale bar: 30 mm. (F) WB analysis of phosphorylated VEGFR2 (Y1175) and total VEGFR2, and phosphorylated Src (Y416) and total Src in HUVECs stimulated with J1ECD ab‐ or IgG ab‐coated beads in the presence and absence of the Src inhibitor Saracatinib. The values were normalized to the IgG bead control. The levels are presented as the mean of each replicate relative to their corresponding control + SEM. All experiments were performed three times. P‐values were obtained with GraphPad Prism after an unpaired t‐test (D), or a two‐way anova with Dunnett's test (B) or Fisher's LSD test (F). Significance is indicated as: ns *p* > 0.05, * *p* < 0.05, ** *p* < 0.01, *** *p* < 0.001, **** *p* < 0.0001. Arbitrary units are indicated as (a.u.).

We next assessed whether direct interaction between Jag1 and VEGFR2 was needed to induce VEGFR2 phosphorylation. Jag1 and VEGFR2 did not interact in co‐immunoprecipitation assays (Co‐IP) (Fig. 5A). It is known that VEGFR2 is activated by the VEGFR2/PECAM/VE‐Cadherin [51] and PlexinD1/NRP1 [50] mechanosensitive complexes. To understand whether proteins involved in these complexes were important for Jag1‐mediated VEGFR2 activation, we used the COS‐7 cell line, which lacks the proteins of these junctional complex sensors of FSS [50, 51]. To our surprise, VEGFR2 was activated by J1ECD ab‐coated beads in the absence of any of the other proteins in the complexes (Fig. 5B), indicating an alternative mode of VEGFR2 activation. Jag1 signaling has been shown to intersect with Src kinase signaling [60, 61, 62], and Src has been shown to operate both downstream and upstream of VEGFR2 [8, 11, 12]. We analyzed Src kinase activity in the COS‐7 cells treated with the J1ECD ab‐coated beads. Jag1 stimulation induced Src and VEGFR2 activity without any other protein of the mechanosensory complexes. Furthermore, Jag1 stimulation induced Src activation even in the absence of VEGFR2 (Fig. 5B), indicating that Src acts upstream of VEGFR2. We found that Src co‐immunoprecipitated with Jag1, indicating a potential direct interaction (Fig. 5C). Proximity ligation assays (PLA) further confirmed close proximity of Src and Jag1 (Fig. 5D,E). To test whether Src activity was necessary for VEGFR2 phosphorylation in endothelial cells, we stimulated HUVECs with J1ECD ab‐coated beads in the presence and absence of the Src inhibitor Saracatinib. Inhibition of Src prevented VEGFR2 phosphorylation, demonstrating that Jag1‐mediated VEGFR2 activation required Src activity (Fig. 5F).

## Discussion

Taken together, our data describe a new noncanonical role of Jag1 in regulating the activity of kinases involved in endothelial mechanotransduction. Physical stimulation of Jag1 induces Src activity, which in turn is required for Jag1‐mediated VEGFR2 activation. Since exposure to J1ECD ab‐coated beads was sufficient to activate Src without requiring a magnetic force, but conjugating the antibody to beads was necessary, we hypothesize that the relocalization and clustering of Jag1 and Src interaction may trigger Src activation, in a similar manner as what has been observed with integrins [53, 54, 55]. The clustering induced by the antibody‐coated beads might be analogous to the relocalization and clustering of Jag1 induced by FSS we observe in endothelial cells exposed to shear stress.

Although Jag1‐mediated VEGFR2 activation appears to be mediated through Src, the underlying mechanism remains unclear. Other groups have previously shown that the cleaved extracellular domain of Jag1 is capable of activating Src in the vasculature by modulating both canonical and noncanonical Notch signaling pathways [60]. However, we demonstrated that Jag1‐mediated VEGFR2 activation does not require Notch activity as VEGFR2 can be activated in the presence of the γ‐secretase inhibitor DAPT that inhibits both canonical and noncanonical Notch activity [18, 58]. Other members of the junctional complex like NRP1, PECAM, or VE‐Cadherin could serve as links between Jag1 and Src, aiding in inducing VEGFR2 activation upon mechanical stress. Nevertheless, the fact that we are able to see Src VEGFR2 activation in COS‐7 cell (Fig. 5B), which do not express any of these proteins [50, 51], indicates they are not required for Jag1‐induced signaling. On the other hand, both Jag1 and Src have a PBM in their structure, which could mediate their interaction through PDZ domain‐containing scaffold proteins regulating Src activity. For example, both Src [63] and Jag1 [64] bind the junctional scaffolding protein Afadin through their PBM. The interaction between Src and Afadin is known to regulate the localization, phosphorylation state, and activity of Src [63]. Afadin has also been shown to regulate VEGFR2 activity [65]. Interestingly, PDZ domains were recently suggested to form mechanosensitive catch bonds with mechanical load regulating PDZ–peptide interactions [66]. FSS‐induced modulation of PDZ‐ mediated interactions may promote Src activity and VEGFR2 phosphorylation. Of note, the junctional protein Neuropilin also harbors a PDZ binding domain required for VEGFR2 [67], further supporting a critical role for PDZ domain mediated interactions in Src and VEGFR2 signaling. However, the exact mechanisms by which Jag1 activates Src warrants further investigation.

Our data could help explain some of the discrepancies between the effects of deleting Jag1 and Notch1 in the endothelium. We observed that Jag1 relocalizes in response to FSS polarizing downstream of the flow direction. Notch has previously been shown to also polarize in the direction of flow. Since cisinteractions with Jag1 inhibit Notch activation [27, 34], our data could explain the contrasting effects of Notch and Jag1 in atherosclerosis [27, 29]. Endothelial dysfunction, induced by low magnitudes of FSS, is characterized by an increase in proliferation, permeability, and impaired endothelial to vascular smooth muscle cell communication leading to the formation of atherosclerotic plaques [1, 5]. Jag1 is induced at low magnitudes of FSS and may act as a cisinhibitor of Notch and an activator of promitogenic pathways downstream of VEGFR2 and ERK. Jag1 and Notch localization downstream of FSS may impair both the ability of Notch to act as an atheroprotective signal in endothelial to endothelial signaling, but also the ability of Jag1 to activate Notch 2 and 3 in vascular smooth muscle cells to maintain their differentiated phenotype [32, 37]. Higher magnitudes of FSS, which decrease Jag1 and increase Notch1 levels in the endothelium (Fig. 1G,H) [29, 30], may serve as a release mechanism for Notch1‐Dll4 signaling in the endothelium, preventing endothelial cell dysfunction and atherosclerotic plaque formation.

While we found Jag1 to be a positive regulator of ERK activity, other groups have shown that Notch is a negative regulator of endothelial ERK activation in mouse and zebrafish [68, 69]. Unlike Notch1 [27], Jag1 and ERK are pro‐atherogenic [29, 70, 71]. Furthermore, as with jag1b deletion, ERK inhibition leads to pericardial edema and defects in outflow tract development in zebrafish [44, 45, 46]. Since we detected pericardial edema in jag1b KO zebrafish without changes in blood flow activity or heart rate (Fig. 3C,D), jag1b loss may affect outflow tract development directly through ERK deregulation. Jag1 regulates outflow tract formation in mice and humans [72, 73, 74], with its expression strongly correlated with outflow tract and coronary vessel development [73]. In zebrafish, flow begins at 25–26 hpf [40] when jag1b is already expressed in the endothelium (Fig. 3B). Expression of jag1 further increases at 2 dpf (Fig. 3B) concomitant with an increase in Notch activity [41]. Outflow tract development occurs in several stages and begins at 24 hpf [75] and is regulated by ERK in zebrafish [76, 77]. Further development to form functional valves at 6 dpf requires Notch signaling [78]. As Jag1b deletion led to pericardial edema but did not alter Notch activity (Fig. 3I–M), our data suggest a potential noncanonical role for Jag1 in this system. Nonetheless, the precise contribution of noncanonical Jag1‐mediated mechanotransduction in cardiovascular physiology and pathology will require further remains to be determined.

## Materials and methods

### Computer fluid dynamics simulation

In this study, to calculate the WSS on cells that attached on the substrate of petri dishes, a computational fluid dynamics (CFD) approach was employed using the software ANSYS‐CFX (ANSYS Inc., Canonsburg, PA, USA). The medium was modeled as an incompressible and Newtonian fluid with a dynamic viscosity (*μ*) of 0.7 mPa·s at 37 °C and a density of 1000 kg·m^−3^ [79]. According to our previous study [79], the turbulent flow (i.e., κ‐epsilon turbulence model) was incorporated into the Navier–Stokes equation. Similar as previous studies [80, 81, 82], the volume of fluid (VOF) technique was used for tracking the medium–air interface during shaking. The loading condition was prescribed on the whole fluid domain (i.e., coordinate O rotated with reference to coordinate O'). The dish wall was rigid with a no‐slip boundary condition. The wall shear stress *τ* on the substrate of the dish was calculated by:

$$\tau =\mu \cdot {\left.\frac{\partial u}{\partial z}\right|}_{z=0}$$

where *u* is the fluid velocity parallel to the substrate and *z* is the distance to the substrate in coordinate O' as shown in Fig. S2.

Using the meshing technique introduced in [79], the fluid domain was meshed by 28 403 hexahedral elements. Transient analysis was used in the simulation with a time step of 0.005 s for a wholesce‐time length of 3.6 s. Three types of loading (i.e., rotation speed = 100 rpm, 150 rpm and 200 rpm) were simulated. Finally, the CFD model was solved by a finite volume method (FVM) using ANSYS CFX under the convergence criteria of the root mean square residual of the mass and momentum < 10^−4^.

In this study, the radial shear stress distribution was normalized with *d* using the method described in [79]. Afterward, oscillatory shear index (OSI), a measurement method for the degree of variation in the shear direction, was identified for each radial position using:

$$\mathrm{OSI}=1-\frac{\left|\int_{0}^{T}\mathrm{WSS}\;\mathrm{dt}\right|}{\int_{0}^{T}\left|\mathrm{WSS}\right|\mathrm{dt}}$$

where *T* is one orbital period. The main shear direction was defined by the direction of the sum of all shear stress vectors at each radial position.

### Cell culture

Pooled primary human umbilical vein endothelial cells (HUVEC) (PromoCell) were cultured in Endothelial Cell Growth Medium 2 (PromoCell) completed with Endothelial Cell Growth Medium 2 SupplementMix (PromoCell). Primary human Aortic Endothelial Cells (HAoEC) (PromoCell) were cultured in Endothelial Cell Growth Medium MV (PromoCell) completed with Growth Medium MV SupplementMix (PromoCell). Thawing and expansion of primary endothelial cells were executed according to the PromoCell Instruction Manual, and all experiments, unless stated otherwise, were done using fully confluent cells at Passages 5–6. COS‐7 cells (RRID:CVCL_0224, ATCC) were cultured in DMEM (Sigma) supplemented with 10% FBS, 100 U·m^−1^ penicillin, and 100 μg·m^−1^ streptomycin. All cells were seeded and expanded on tissue culture polystyrene (TCPS) plates and maintained at 37 °C, 5% CO_2_. All experiments were performed with mycoplasma‐free cells.

### Experimental animals

This study complies with the EU directive 2010/63/EU on the protection of animals used for scientific purposes and equivalent Finnish National Legislation, the Act on the Protection of Animals Used for Scientific or Educational Purposes (497/2013), according to which vertebrate animals are not considered ‘protected animals’ until they reach the stage of independent feeding which typically occurs for zebrafish around 5 dpf. All experiments involving zebrafish used embryos at 4 dpf and therefore no further ethical approvals were required.

Jagged1b (b1105) [83] was generously provided by the Crump Lab at the University of Southern California, Keck School of Medicine. All the experiments were carried out at Turku Bioscience Center Zebrafish Core under license ESAVI/31414/2020 and ESAVI/44584/2023 granted by The Regional Administration Office of Southern Finland. jag1b ± strain and TP1:H2B‐mCherry; jag1b ± strain were created in Turku by crossing jag1b ± together with tg(*TP1:H2B‐mCherry*) strain [49] generously provided by the Ninov Lab at the Center for Regenerative Therapies Dresden (CRTD) at TUD Dresden University of Technology. Embryos used for imaging were kept in E3 media (5 mm NaCl, 0.17 mm KCl, 0.33 mm CaCl_2_, 0.33 mm MgSO_4_) supplemented with 30 mg·m^−1^ of 1‐phenyl 2‐thiourea (PTU) in 28.5 °C. Imaging was carried out using Zeiss AxioZoom V.16 stereomicroscope with 1.0 × Plan ApoZ (NA 0.125) objective and Hamamatsu sCMOS ORCA‐Flash4.0 LT. During the imaging, embryos were anesthetized with Tricaine 160 mg·m^−1^. Notch activity was measured using ImageJ FIJI image analysis software. For western blot (WB) analysis, embryos were genotyped and then pooled accordingly. Embryos were lysed with 3 × Laemmli buffer (30% Glycerol, 3% SDS, 0.m Tris–HCl pH 6.8, 0.015% bromophenol blue, 3% β‐mercaptoethanol) and boiled 15 min at 95 °C.

For genotyping, the genomic DNA was extracted from each embryo via alkaline lysis using 50 mM NaOH and boiling the samples for 15 min at 95 °C. Samples were vortexed every 5 min and at the end centrifuged 5 min, 14 000 relative centrifugal force (rcf) at 4 °C. DreamTaq DNA polymerase (Thermo Scientific) was used in gene amplification according to the manufacturer's instructions followed by BseGI (BtsCI) (Thermo Fisher Scientific) restriction enzyme treatment. Following primers were used in gene amplification: Forward primer: GTACCAAATCCGGGTGACCT, Reverse primer: GTGGCTTTTTGGGTCATTATCA. Samples were analyzed from 2.5% Agarose gel. BseGI treatment resulted fragments listed below.

**Table:** 

jag1b
bp
+/+: 134, 72
+/−: 206, 134, 72
−/−: 206

### Shear stress experiments

For imaging experiments, HUVECs and HAoECs were seeded into 6‐channel slides (Ibidi, 80 606) coated with 100 μg / ml Bovine Type I Collagen solution (Advanced BioMatrix Inc., 5010) with 1 × 10^5^ cells added per channel. Media was changed twice a day. Cells were allowed to attach and form a monolayer overnight, after which they were subjected to shear stress or maintained as static control. Flow over cells was achieved by assembling a perfusion set consisting of the 6‐channel slide connected to a glass bottle with 25 mL of respective culture media via 1.6‐mm inner diameter silicone tubing (Ibidi®, 10 842) and attached to a REGLO Analog peristaltic pump (Ismatec) via three‐stop‐tubing, creating a loop for media recirculation during the experiment. Utilizing a three‐port screw cap on the glass bottle, a 22 μm pore diameter syringe filter was attached to the bottle to allow air pressure equilibration in the system. At the start of a shear stress experiment, the flow rate of the system was increased in a step‐wise manner over 1 h by changing the pump rpm, starting from 0 rpm, followed by 22, 66, and 99 rpm steps, 99 rpm equaling ~ 1 Pa of laminar and continuous FSS. Cells were exposed to flow for 24–25 h at ~ 1 Pa. The media was preconditioned to the temperature, humidity, and CO_2_ conditions of the experiment by putting it in a glass bottle inside the incubator overnight before the assembly of the perfusion set.

For gene expression experiments, HUVECs were seeded at 10^6^ cells·ml^−1^ into collagen IV‐coated 1‐channel slides for gene expression analysis and 6‐channel slides (Ibidi, 80 172) for immunocytochemistry or the reporter cell assay. The next day, endothelial cells were exposed to FSS using the Ibidi pump system (Ibidi) or maintained without any additional mechanical stress as static controls. The standard perfusion sets were adjusted to gain a stable flow speed and pressure. Resistance tubing (0.5 mm inner diameter) was added after the channel slides to stabilize the fluctuations in flow speed. The cells were exposed to 0.5, 1, or 2 Pa FSS for 24 h. Flow speeds were constantly measured to ensure correct flow speed (ME2PXL flow sensor, Transonic Systems Inc.).

For protein and phosphorylation analysis, cells were seeded in six‐well plates and grown to full confluency. Before flowing, in the beginning of the experiment, the media was changed to ensure an initial volume of 3 mL per well and the plate was placed on a CO_2_‐resistant orbital shaker. A computational fluid dynamics simulation was performed as previously described [79] for a 19 mm shaking orbital diameter. The simulation values were used to select the speed of the orbital shaker, and in all cases, a nonflowed plate (Static) was used as a control. Unless stated otherwise, plates were collected after 24 h and placed on ice, and approximately 64% of the outermost area of the well was collected.

### Immunocytochemistry

After the shear stress experiment, cells in the 6‐channel Ibidi® slides were washed two to three times with 37 °C modified (without CaCl_2_, MgCl_2_) Dulbecco's Phosphate‐Buffered Saline (DPBS) and fixed in 37 °C 4% paraformaldehyde in DPBS for 10 min, followed by another three washes with modified DPBS. Slides with fixed cells were stored for up to 16 days at a temperature of 4 °C in the dark. Cells were permeabilized in 0.2% Triton X‐100 in DPBS for 5 and 10 min at room temperature (RT), after which they were washed once with modified DPBS. Blocking of the cells was done by incubation in 1% BSA in modified DPBS at RT for 30 min, followed by three washes with modified DPBS. Cells were incubated with Jag1 (Cell Signaling Technology; 2620; 1 : 100) and CD31 (Invitrogen; 37–0700; 1 : 100) monoclonal primary antibodies in the solution of 3% BSA and 0.05% Triton X‐100 in DPBS overnight at 4 °C. Cell was washed three times with modified DPBS and incubated with secondary antibodies (Donkey anti‐Mouse IgG Alexa Fluor 488; A‐21202; 1:500, Goat anti‐Rabbit IgG Alexa Fluor 555; A‐21428, 1 : 500) and DAPI (Sigma‐Aldrich, D9542) in 3% BSA and 0.05% Triton X‐100 in PBS for 1 h at RT in the dark. The staining solution was removed, and cells were washed three times in RT‐modified DPBS and imaged with the channels filled with PBS.

Imaging was conducted with a Zeiss LSM 880 Airyscan confocal with an Axio Observer.Z1 microscope using zen 2.3 SP1 black edition acquisition software. The objective used was 63 × Zeiss C Plan‐Apochromat Oil DIC M27 with 1.4 aperture. The pinhole was set to give the same optical section thickness for all channels. DAPI was excited with a diode at a wavelength of 405 nm and acquired at an emission window of 410–514 nm with a PMT with the pinhole set to 3.85 airy units (AU). Alexa Fluor 488 was excited with Argon laser at a wavelength of 488 nm and acquired at an emission window of 490–579 nm with GaAsP detector with pinhole set to 3.10 AU. Alexa Fluor 555 was excited with HeNe laser at a wavelength of 543 and acquired at an emission window of 556–648 with a cooled PMT with the pinhole set to 2.69 AU. Channels were acquired sequentially and with unidirectional scanning, 4 times line averaging, and pixel dwell time of 1.02 μs at a bit depth of 8 bits. All images were acquired with 1 × 1 binning, and the XYZ pixel dimensions varied across experiments within 85–132 nm in X and Y and 632–1090 nm in Z.

PolarityJaM [34] was used to quantitatively measure the polarization of Jag1 from confocal images. To preprocess the confocal 3D images for polarityjam, two channel 2D images were generated from the confocal 3D image stacks by maximum intensity projection of the Jag1 channel and by picking optical section corresponding to the base of the cell from PECAM‐1 channel. In PolarityJaM, PECAM‐1 channel was used as the junction channel to generate cell masks and signal polarity was measured from Jag1 channel. PolarityJaM parameters used in the analysis will be available in the repository upon acceptance for publication.

### Proximity ligation assay (PLA)

HUVECs were grown until fully confluent on glass bottom ibidi 8 well chamber slides. Cells were fixed in 37 °C 4% paraformaldehyde in DPBS 4% PFA for 15 min and permeabilized with 0.3% Triton‐X100 in DPBS for 10 min. Samples were blocked and stained as described in the duolink PLA Fluorescence Protocol from Merck. The antibodies used were Jagged1 28H8 from CST and Src GD11 from abcam with duolink *In Situ* PLA® Probe Anti‐Rabbit PLUS and duolink *In Situ* PLA® Probe Anti‐Mouse PLUS respectively. After finishing the protocol, samples were maintained in ibidi mounting medium and store 4 °C until imaging.

Imaging was conducted with a Leica STELLARIS 8 FALCON FLIM with FCS and Resonant Scanner confocal laser scanning microscope using Leica's LAS X acquisition software. The objective used was HC PL APO 63 ×/1.40 OIL CS2. DAPI was excited with a wavelength of 405 nm and acquired at an emission window of 430–529 nm with Power HyD X detector in digital operating mode. The texas red PLA signal was excited with a wavelength of 593 nm and acquired at an emission window of 598–640 nm with Power HyD S detector in analof operating mode. Channels were acquired sequentially with unidirectional scanning and line averaging of 2. Pixel dwell time was 0.775 μs at a bit depth of 16 bits. All images were acquired with 1 × 1 binning. Image voxel dimensions in X and Y varied between experiments from 60 nm to 160 nm isometrically, and in Z dimension were 300 nm across all experiments.

Quantification was obtained using a CellProfiler pipeline consisting of an IdentifyPrimaryObjects module to determine the amount of PLA signals per field of view and subsequently the number of signals mm^−2^. Images were preprocessed in Image J to remove the same amount of background signal from every image and store each channel separately for analysis in cellprofiler. The macro used for preprocessing as well as the cell profile pipeline are available in the repository. The number PLA signals per area was calculated for every image and used for statistical analysis in GraphPad Prism.

### Co‐immunoprecipitations and Western blots (WB)

Cells were collected in lysis buffer (50 mm Tris, pH 7.5, 150 mm NaCl, 1% Triton X‐100, 0.1% SDS) with protease (Complete™ Protease Inhibitor Cocktail, Merck) and phosphatase inhibitor cocktail (Pierce™ Phosphatase Inhibitor Mini Tablets, ThermoFisher). Lysates were collected and kept on ice during sonication before being centrifuged for 10 min at 14 000 rcf at 4 °C. The supernatant was used for the input control and the subsequent processing steps. Beads were washed five times with washing buffer (50 mm Tris–HCl (pH 7.5); 250 mm NaCl; 0,1% NP‐40) before use. Lysates were precleared using 5 μL of washed magnetic beads per sample. After preclearing, samples were incubated overnight at 4 °C with antibody (Jag1 28H8 1:50, VEGFR2 55B11 1:100 or IgG (DA1E); all from Cell Signaling Technologies (CST)). Samples were then incubated at RT for 1.5 h with 16.5 μL of washed beads per sample. Finally, beads were washed three times with a washing buffer and, one final time, with MilliQH_2_O before being diluted in Laemmli sample buffer for WB.

Proteins were separated by SDS/PAGE and transferred to a Protran nitrocellulose membrane (GE Healthcare Life Sciences) using a wet transfer apparatus (Amersham Bioscience). The membranes were blocked with 5% nonfat dry milk in TBS at RT for 0.5–1 h. Primary antibody incubation was done overnight at 4 °C in constant agitation. Membranes were then incubated in secondary antibody for 1 h at RT (1 : 10000). Proteins were acquired using an iBright Imaging System (ThermoFisher) after incubation with SuperSignal West Pico PLUS Enhanced chemiluminescence substrate (ThermoFisher). The following antibodies were used: Jag1 28H8, VEGFR2 55B11, phospho‐Tyr1175 VEGFR2 2478, p44/42 MAPK (Erk1/2) 9102S, phospho‐Y204‐Erk1/phospho‐Y187‐Erk2 5726S, Akt 9272, phospho‐Ser473 Akt 4060S, CD31 (PECAM‐1) D8V9E, VE‐Cadherin D87F2, Src 2108S, and Phospho‐Src Family (Tyr416) D49G4 all purchased from CST. HSC70 (ADI‐SPA‐815‐D, Enzo) or Revert 700 Total Protein Stain (LI‐COR) was used for loading control. The densitometry level were obtained using the image analysis software ImageJ FIJI.

### Magnetic bead experiments

Experiments were performed using 24‐well plates. 1.0 μm Protein A/G Magnetic Beads (ThermoFisher) was washed with TBS and coated for 1.5 h at RT with antibodies (Jag1 1C4, IgG DA1E from CST; Notch 2 ECD MA5‐24274 from ThermoFisher) or recombinant peptides (Recombinant Human Notch‐1 Fc chimera protein and IgG‐Fc chimera protein from R&D systems). Cells were incubated with beads at 37 °C, 5% CO_2_ for 15 min before exposure to a magnetic field using permanent magnets for another 15 min or were incubated for 30 min before being lysed and collected for WB analysis.

### Pharmacological inhibitions

HUVECs were treated with the γ‐secretase inhibitor 1 N‐[N‐(3,5‐difluorophenacetyl)‐l‐alanyl]‐S‐phenylglycine t‐butyl ester (DAPT) to inhibit Notch cleavage or CB‐103 to inhibit Notch‐induced transcriptional activation, both at 25 μm. To inhibit Src, HUVECs were incubated with the inhibitor Saracatinib at 10 m. All inhibitors were added in fresh media, 24 h before experiments were performed. Inhibitors were dissolved in DMSO, and control experiments were performed in the presence of the same amount of DMSO as vehicle control.

### Gene expression

Performed as previously described by our group [31]. RNA was isolated with the Qiagen RNeasy kit. The β‐mercaptoethanol–RLT buffer mixture was directly added to the cells in the 1‐channel slides. The synthesis of cDNA was performed with M‐MLV reverse transcriptase (Invitrogen). For six reference genes tested GAPDH was the most stably expressed, as analyzed with GeNorm130. The PCR protocol consisted of 3 min at 95 °C, followed by 40 cycles of 20 s at 95 °C, 20 s at 60 °C and 30 s at 72 °C. Data were analyzed using the ΔΔCt method. The primers used can be found in Table 1.

**Table 1:** Primer list. List of primers used for gene expression analysis.

Gene	Forward	Reverse
NOTCH1	CCTGAAGAACGGGGCTAACA	GATGTCCCGGTTGGCAAAGT
NOTCH3	GCCATGCTGATGTCAATGCT	CAGCCCAGTGTAAGGCTGAT
NOTCH4	TGCCAGCCCAAGCAGATATGTA	CCAACCCACGTCACACACAC
DLL1	ATTGACGAGTGTGACCCCAG	GCACAGGTCATGGCACTCAA
DLL4	CCTCTCCAACTGCCCTTCAAT	GCGATCTTGCTGATGAGTGC
JAG1	AATGGCTACCGGTGTGTCTG	CCCATGGTGATGCAAGGTCT
HES1	AGTGAAGCACCTCCGGAAC	CGTTCATGCACTCGCTGA
HEY1	TGGATCACCTGAAAATGCTG	CGAAATCCCAAACTCCGATA
HEY2	TTTGAAGATGCTTCAGGCAA	GGCACTCTCGGAATCCTATG

### Notch reporter assay

Performed as previously described by our group [31]. Notch1 overexpressing HEK293T cells were transfected with 12 × CSL‐luciferase223 or GFP as a transfection control using polyethyleneimine (PEI), 1 mg·m^−1^. For transfection, DNA and PEI were mixed at 1 : 2 weight ratio in plain medium. After 5 min incubation, the DNA‐PEI mixture was added to the HEK293T cell culture. Next day, the cells were washed with PBS and used for the reporter assay. After flow experiments, endothelial cells were washed twice with PBS. Transfected HEK293T cells were seeded directly on top of endothelial cells (10^6^ cells·ml^−1^, 50 μL/channel) and cultured in EGM2 medium for 24 h. In half of the channels (3), the cells were cocultured in the presence of 50 μm 6‐alkynyl‐fucose (Peptides International). In the other channels, the cells were cultured in an equal amount of DMSO as a control. Notch activity was assessed by measuring luciferase activity from lysed cells using Luciferase Assay from Promega. Biotek Synergy plate reader was used in signal detection.

### Statistical analysis

*P*‐values were obtained after parametric tests were conducted. For two group comparisons a two‐tailed Student's *t*‐test was performed. When three or more comparisons or more than one independent variable were present, two‐way ANOVA were performed to assess main effects, with post‐hoc testing. Dunnet's post‐hoc test was used when groups were compared with normalizing control. Tukey's post‐hoc test was used when all groups where compare with each other. Bonferroni correction was performed when four or more preselected comparisons against controls were made. For four or less comparisons, Fisher's LSD test was used as the post‐hoc test. All error bars represent the SEM. The graphs were made using an established color‐blind friendly palette [84]. Graph and statistical analysis were performed using the statistical software graphpad Prism 10.

## Author contributions

FSR and CMS were involved in conceptualization. RCHD, FZ, and OMJAS were involved in methodology. FSR, NV, EK, RCHD, FZ, and OMJAS were involved in investigation. FSR, NV, EK, RCHD, FZ, OMJAS, and CMS were involved in formal analysis. CVCB and CMS were involved in resources. OMJAS and FSR were involved in data curation. FSR and CMS were involved in writing—original draft. FSR, NV, EK, RCHD, FZ, CVCB, OMJAS, and CMS were involved in writing—review & editing. FSR was involved in visualization. CVCB, OMJAS, and CMS were involved in supervision. FSR and CMS were involved in project administration. CVCB and CMS were involved in funding acquisition.

## Funding

This project has received funding from the following sources: European Research Council (ERC) ERC‐CoG No 771168 (ForceMorph). European Research Council (ERC) ERC‐SynG No 101167065 (Making Blood). Research Council of Finland, Center of Excellence, decision number #374179 (CoEIMMENs). Research Council of Finland, decision number #316882 (SPACE). Research Council of Finland, decision number #330411 (SignalSheets). Research Council of Finland, decision number #336355 (Solutions for Health at Åbo Akademi University). Research Council of Finland, decision number #337531 and #357911 (InFLAMES Flagship Program). Åbo Akademi University Foundation's Centers of Excellence in Cellular Mechanostasis (CellMech) and Bioelectronic Activation of Cell Functions (BACE). The work performed by FSR has been partially funded by personal grants from The Swedish Cultural Foundation in Finland, Instrumentarium Science Foundation and Magnus Ehrnrooth Foundation.

## Conflicts of interest

The authors declare no conflicts of interest.

## Supporting information


**Fig. S1.** Validation of Jag1 ICD and ECD antibodies.
**Fig. S2.** Mesh of the CFD model geometry.

## Data Availability

All data, code, and materials used in the analysis are available in the 4TU.ResearchData repository at https://doi.org/10.4121/47b050fe‐bf78‐4aed‐b7a4‐38d06d3ddb9a or available upon request.
