# Supplementary material for: A noncanonical role for Jagged1 in endothelial mechanotransduction
Source: FEBS J. 2026 Feb 23;293(15):4604–23. doi: 10.1111/febs.70466 (PMC13440619; doi:10.1111/febs.70466)
Supplement: Supplementary file 1 [file FEBS-293-4604-s001.pdf]

A non-canonical role for Jagged1 in endothelial mechanotransduction

Freddy Suarez Rodriguez, Noora Virtanen, Elmeri Kiviluoto, Rob C. H. Driessen, Feihu Zhao, Carlijn V. C. Bouten, Oscar M. J. A. Stassen, Cecilia M. Sahlgren

Supplementary figures

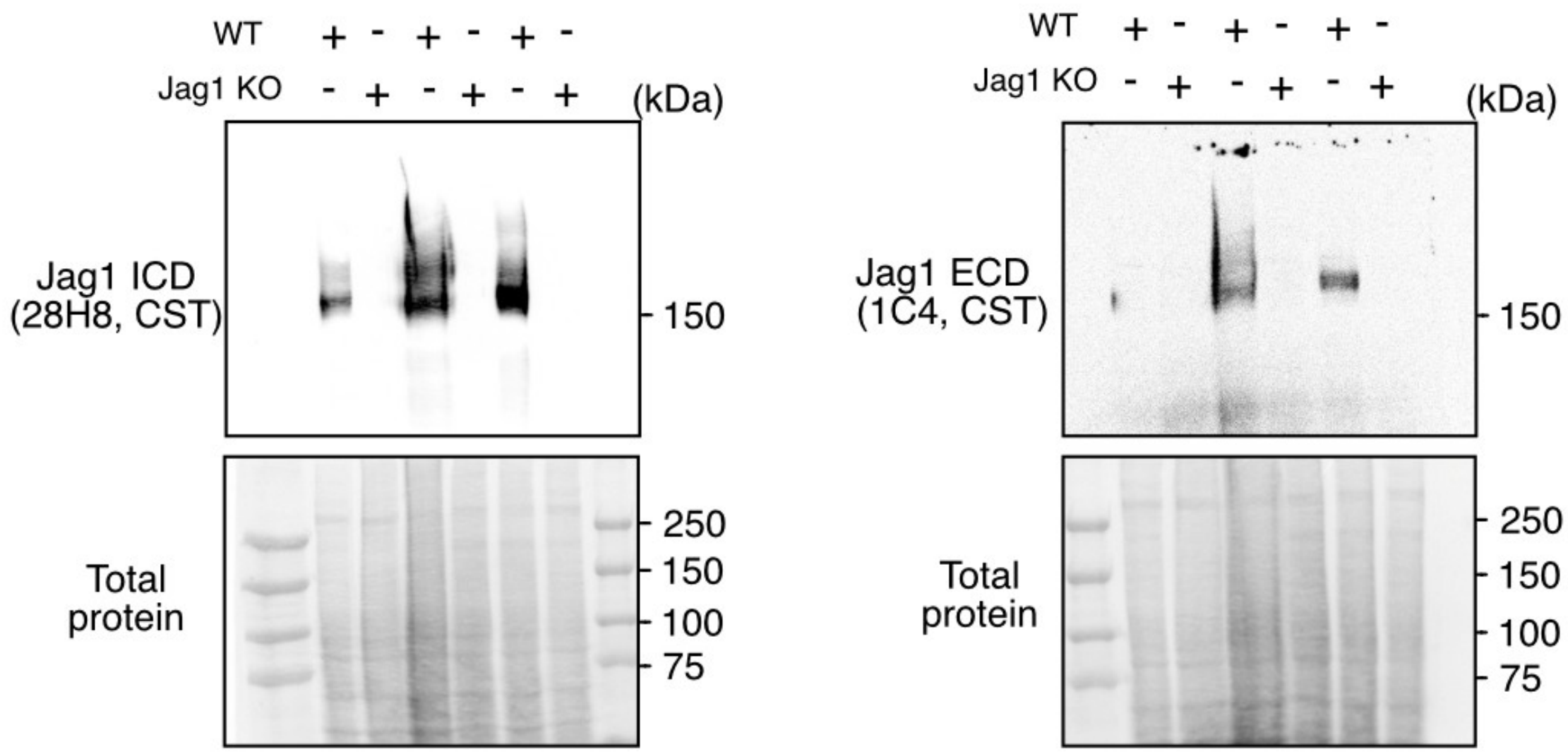

fig. S1. Validation of Jag1 ICD and ECD antibodies. Western blot of Jag1 knockout (KO) or WT BON1 cells with the following antibodies: Jag1 28H8 antibody from cell signaling technologies (CST) which recognizes Jag's intracellular domain (ICD) and Jag1 antibody 1C4 from CST, which recognizes Jag's extracellular domain (ECD). Representative blots from three independent experiments are shown. Both antibodies recognize Jag1 full length (180 kDa) specifically. We observe an unexpected unspecific signal around 50 kDa with the Jag1 1C4 antibody in both WT and Jag1KO. A similar pattern is observed in the total protein stain, indicating a potential bleed-through due to the weaker signal from the Jag1 1C4 antibody compared to that of the Jag1 28H8 antibody.

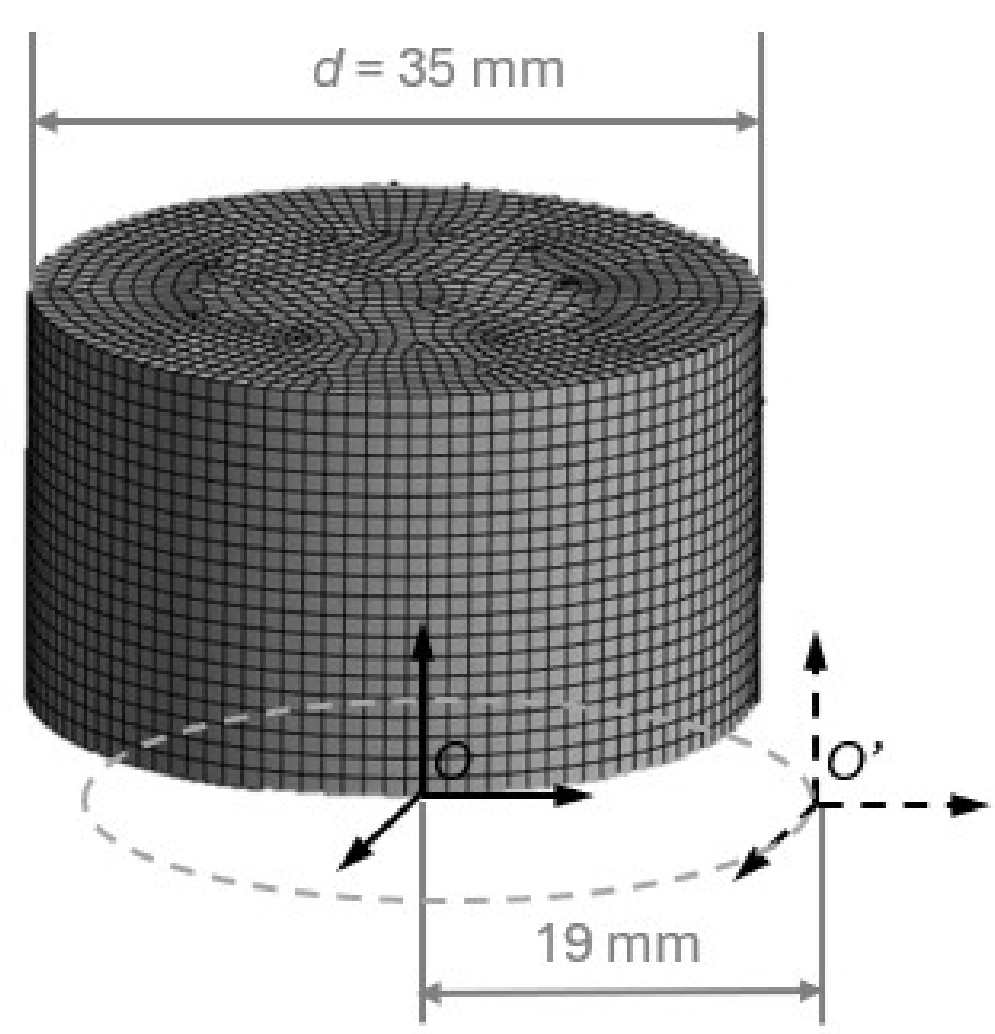

fig. S2. Mesh of the CFD model geometry. Mesh of the computational fluid dynamics simulation (CFD) of a plastic dish obtained using the software ANSYS-CFX (ANSYS Inc., Canonsburg, PA, USA). Diameter is indicated as d. O indicates the center of the dish and O' the reference coordinate.
